# 90Y SPECT scatter estimation and voxel dosimetry in radioembolization using a unified deep learning framework

**DOI:** 10.1186/s40658-023-00598-9

**Published:** 2023-12-13

**Authors:** Yixuan Jia, Zongyu Li, Azadeh Akhavanallaf, Jeffrey A. Fessler, Yuni K. Dewaraja

**Affiliations:** 1 Department of Electrical Engineering and Computer Science, University of Michigan, 4125 EECS Bldg., 1301 Beal Ave., Ann Arbor, MI 48109 USA; 2 Department of Radiology, University of Michigan, Ann Arbor, MI USA

**Keywords:** 90Y, SPECT, Radioembolization, Scatter correction, Dosimetry, Deep learning

## Abstract

**Purpose:**

90Y SPECT-based dosimetry following radioembolization (RE) in liver malignancies is challenging due to the inherent scatter and the poor spatial resolution of bremsstrahlung SPECT. This study explores a deep-learning-based absorbed dose-rate estimation method for 90Y that mitigates the impact of poor SPECT image quality on dosimetry and the accuracy–efficiency trade-off of Monte Carlo (MC)-based scatter estimation and voxel dosimetry methods.

**Methods:**

Our unified framework consists of three stages: convolutional neural network (CNN)-based bremsstrahlung scatter estimation, SPECT reconstruction with scatter correction (SC) and absorbed dose-rate map generation with a residual learning network (DblurDoseNet). The input to the framework is the measured SPECT projections and CT, and the output is the absorbed dose-rate map. For training and testing under realistic conditions, we generated a series of virtual patient phantom activity/density maps from post-therapy images of patients treated with 90Y-RE at our clinic. To train the scatter estimation network, we use the scatter projections for phantoms generated from MC simulation as the ground truth (GT). To train the dosimetry network, we use MC dose-rate maps generated directly from the activity/density maps of phantoms as the GT (Phantom + MC Dose). We compared performance of our framework (SPECT w/CNN SC + DblurDoseNet) and MC dosimetry (SPECT w/CNN SC + MC Dose) using normalized root mean square error (NRMSE) and normalized mean absolute error (NMAE) relative to GT.

**Results:**

When testing on virtual patient phantoms, our CNN predicted scatter projections had NRMSE of 4.0% ± 0.7% on average. For the SPECT reconstruction with CNN SC, we observed a significant improvement on NRMSE (9.2% ± 1.7%), compared to reconstructions with no SC (149.5% ± 31.2%). In terms of virtual patient dose-rate estimation, SPECT w/CNN SC + DblurDoseNet had a NMAE of 8.6% ± 5.7% and 5.4% ± 4.8% in lesions and healthy livers, respectively; compared to 24.0% ± 6.1% and 17.7% ± 2.1% for SPECT w/CNN SC + MC Dose. In patient dose-rate maps, though no GT was available, we observed sharper lesion boundaries and increased lesion-to-background ratios with our framework. For a typical patient data set, the trained networks took ~ 1 s to generate the scatter estimate and ~ 20 s to generate the dose-rate map (matrix size: 512 × 512 × 194) on a single GPU (NVIDIA V100).

**Conclusion:**

Our deep learning framework, trained using true activity/density maps, has the potential to outperform non-learning voxel dosimetry methods such as MC that are dependent on SPECT image quality. Across comprehensive testing and evaluations on multiple targeted lesions and healthy livers in virtual patients, our proposed deep learning framework demonstrated higher (66% on average in terms of NMAE) estimation accuracy than the current “gold-standard” MC method. The enhanced computing speed with our framework without sacrificing accuracy is highly relevant for clinical dosimetry following 90Y-RE.

**Supplementary Information:**

The online version contains supplementary material available at 10.1186/s40658-023-00598-9.

## Introduction

Radioembolization (RE) with microspheres radiolabeled with 90Y, a *β*-emitter, is a treatment option for inoperable primary or metastatic liver cancer [1]. In recent years, the importance of accurate post-therapy imaging-based dosimetry for RE treatment verification and for establishing dose–response relationships for use in dosimetry-guided treatment planning in future patients has been well recognized [2]. Furthermore, the concept of using 90Y absorbed dose maps to plan subsequent treatment with external beam radiotherapy (EBRT) to under-dosed regions is being explored [3, 4]. Post-therapy dosimetry based on directly imaging the delivered 90Y instead of pre-therapy imaging with surrogates such as 99mTc Macro Albumin Aggregated (99mTc-MAA) is preferred for such application due to differences between predicted and delivered activity distributions.

Treatment with 90Y offers the potential for both therapy and quantitative imaging. However, lacking gamma-ray photons, imaging of 90Y typically relies on SPECT/CT imaging of bremsstrahlung X-rays [5, 6] associated with the *β* decays (*E*_max_ = 2.3 MeV, $\overline{E}$ = 0.94 MeV, mean tissue penetration 2.5 mm, maximum 11 mm) [7]. Alternatively, time-of-flight 90Y PET imaging via a very low abundance positron decay of 90Y (positron yield ~ 3.2 × 10^−5^) [7] has the advantage of superior resolution but suffers from a high noise level [8]. Furthermore, due to the wider accessibility and lower cost of SPECT compared with PET, its application in 90Y-RE imaging is expected to continue and thus should be improved. The challenge of quantitative 90Y SPECT is associated with the continuous bremsstrahlung energy spectrum that extends up to 2.3 MeV. This impacts the spatial resolution because medium or high energy collimation must be used to reduce septal penetration of high energy photons. Furthermore, there are significant downscatter events that contaminate the main window acquisition, limiting quantitative accuracy. Wang et al. studied the multi-modal treatment of hepatocellular carcinoma using 90Y-RE combined with EBRT and identified the poor quantitative accuracy of bremsstrahlung SPECT, performed without scatter correction, as the main limitation for their approach since the SPECT image quantification forms the basis for 90Y dose calculation [3].

Accurate scatter correction is a prerequisite for accurate quantitative SPECT imaging. Clinical systems commonly use simple energy window-based scatter estimation methods such as dual or triple energy window approaches. However, for bremsstrahlung SPECT imaging, such methods are less effective because of the continuous energy spectrum, even though empirical window-based methods have been proposed [9]. When window-based methods are unsuitable or less precise, Monte Carlo (MC) transport algorithms are preferred. MC algorithms fully track particle interactions from their initial points within the patient body to the camera crystal, making them the gold standard for scatter estimation [5, 6]. Nevertheless, the higher computational demands of MC can limit its routine clinical use. In addition to scatter, limited spatial resolution is another physical effect that degrades SPECT quantification accuracy, especially when medium or high energy collimation is used. To reduce the impact of poor spatial resolution and the associated blurring of 90Y SPECT images, reconstruction with collimator detector response (CDR) modeling is used, but this leads to edge-artifacts while de-blurring is still incomplete. Resolution effects are also reduced with partial volume correction (PVC), often in the form of recovery coefficients applied when quantifying mean activity in a target volume. However, voxel-level PVC is a challenging and yet unresolved problem [10].

Following quantitative imaging, the final step in 90Y-RE voxel dosimetry is converting the activity map into an absorbed dose map. Sequential imaging/co-registration to determine pharmacokinetics and time-activity fitting is not required for this therapy because the microspheres are trapped and do not redistribute [2]. Direct MC radiation transport starting with the patient’s hybrid images (for example, activity map from SPECT and density map from CT) is generally accepted as the gold standard for voxel dosimetry; however, it is computationally expensive for routine clinical utilization. Hence simplified models, such as dose voxel kernel (DVK) convolution [11] are widely used, but introduce some additional errors on the estimated dose, particularly in the presence of heterogeneous medium (e.g., lung-liver and bone-marrow interfaces). Despite the superiority of direct MC radiation transport over DVK-based dose estimation, both are derived from the activity map corresponding to the measured emission image; hence, both suffer from the limitations of SPECT described above.

Machine learning algorithms, in particular deep learning, have rapidly advanced in medical image analysis over the past decade [12] and have recently entered the nuclear medicine arena [13–15]. For SPECT scatter estimation, our group proposed a deep convolutional neural network and demonstrated results comparable to MC-based scatter estimation, but at a fraction of the computation cost [16]. For dosimetry, there are a few recent studies that have applied deep learning for both diagnostic (18F and 68Ga) and theranostic (177Lu) radiopharmaceuticals [17–19], although, to our knowledge, not for 90Y therapy. Despite the promising performance, there is an inherent limitation in the training process of these previously proposed dosimetry networks. This problem stems from the fact that the ground truth images for training the networks were generated from SPECT or PET images, hence suffer from the limitations of the modality such as poor resolution, noise, scatter, and reconstruction artifacts. To address this problem for 177Lu dosimetry, we previously proposed a deep residual convolutional neural network that used virtual computational phantoms with a high spatial resolution for the training process [20].

Motivated by the recent interest in accurate 90Y voxel dosimetry and building on our previously implemented CNNs [16, 20], we developed a unified framework that aims to estimate the voxel-level dose-rate map accurately and efficiently, starting with the measured SPECT projections and CT images as input. Our framework consists of (1) a deep learning model for 90Y-SPECT/CT scatter estimation; (2) SPECT reconstruction with the deep learning-based scatter estimate; (3) a residual learning network for 90Y dose estimation, trained using high-resolution virtual phantoms. We trained/validated/tested our framework using a population of virtual patient phantoms relevant to 90Y-RE and compared performance with dosimetry performed using non-learning methods, MC and DVK convolution.

## Materials and methods

**Fig. 1:**
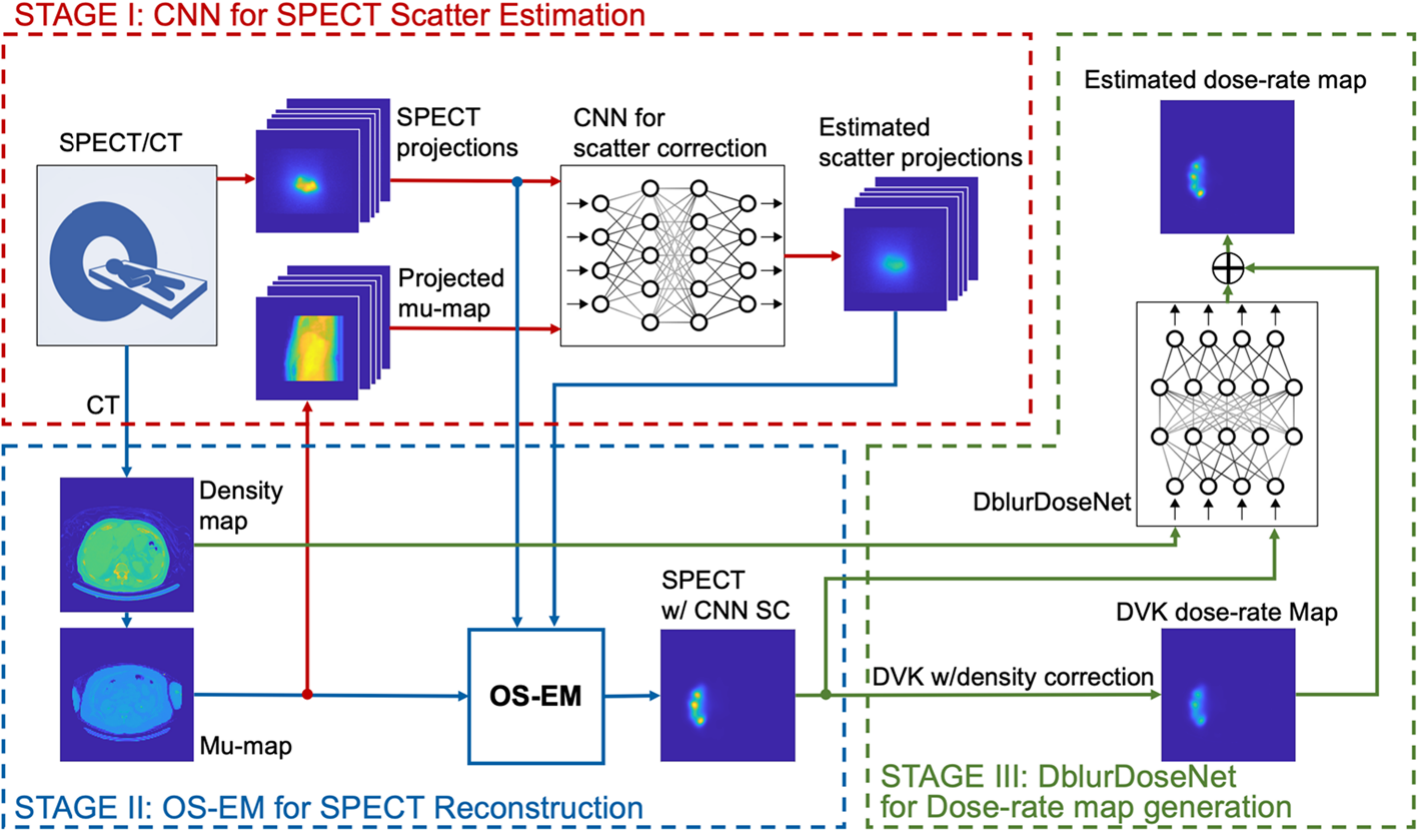
Overview of deep learning framework for 90Y Bremsstrahlung SPECT scatter estimation and voxel dosimetry. Our framework consists of (1) a deep learning model for 90Y-SPECT/CT scatter estimation; (2) SPECT reconstruction with the deep learning-based scatter estimate; (3) a residual learning network for 90Y dose estimation, trained using high-resolution virtual phantoms

Figure 1 presents an overview of the framework, encompassing three main stages: scatter estimation, SPECT reconstruction and voxel-level dosimetry.

### Virtual patient phantom generation

90Y SPECT/CT data for 18 patients who underwent 90Y-RE at our clinic were available for generation of virtual patient phantoms. The lesion contours defined manually by a radiologist and healthy liver (liver minus lesions) contours defined using automated tools were also available. The lesions covered a range of lesion-to-background uptake ratios, shapes, and sizes (median: 26.9 mL, range 5.7–932.1 mL) and represented both left and right lobe treatment. The patient SPECT data were reconstructed with in-house developed ordered-subsets expectation–maximization (OS-EM) software with attenuation, scatter correction and resolution recovery to define virtual patient (digital phantom) activity maps. Note that for the scatter estimation, we used the previous CNN described in [15]. The corresponding density maps were generated from the patient's CT using an experimentally derived calibration curve for CT-to-density conversion. All activity maps and density maps used for simulation were registered to CT image space (matrix size: 512 × 512 × 194, voxel size in mm: 0.98 × 0.98 × 2). Phantoms were divided into training/validation/testing sets for both networks to represent a range of activity distributions and patient sizes (see Additional file 1: Figs. S1–S3 and Additional file 1: Table S1).

### Stage I: convolutional neural network-based estimation of SPECT scatter projections

#### Datasets

We used MC-simulated SPECT projections from 6 virtual patients (6 × 128 projection views) to train and validate the scatter estimation CNN in the first stage, while the rest of 12 virtual patients were used for testing. To train/validate the network, we generated the ground truth (GT) scatter projections by running the SIMIND MC simulation code [21] that couples the digital phantoms with the SPECT/CT camera model based on parameters from our clinic (scanner: Siemens Intevo with HE collimators, crystal size: 5/8″, acquisition window: 105–195 keV, number of projection views: 128, matrix size: 128 × 80, pixel size in mm: 4.8 × 4.8). Approximately one billion photon histories were simulated per projection angle to generate data with low statistical noise. We kept only the central slices of each projection that were within the FOV, reducing the initial projection matrix size of 128 × 128 to 128 × 80 for each view angle. Poisson noise was added after scaling projections to count levels observed in the clinic, ~ 10^7^ counts over all views.

#### Network

**Fig. 2:**
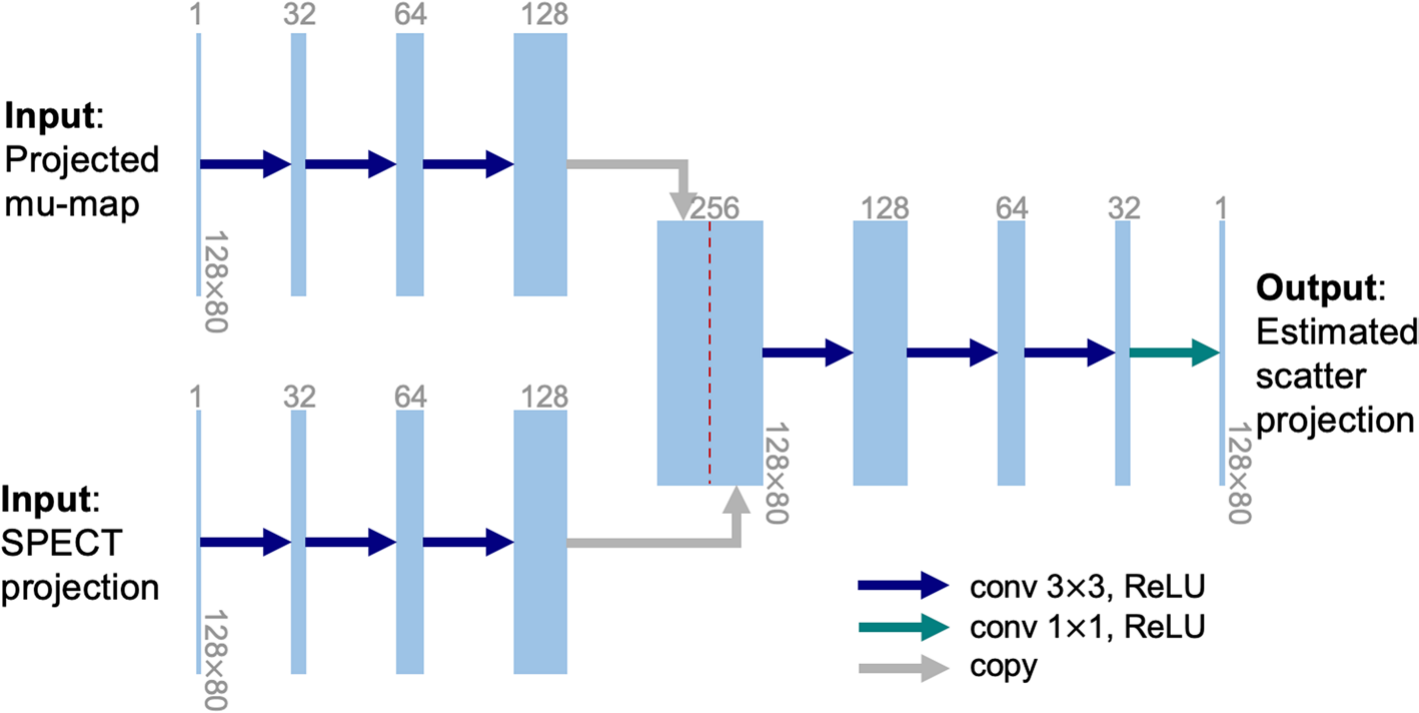
Architecture of CNN for 90Y Bremsstrahlung scatter estimation in SPECT projection space. Each blue box corresponds to a multi-channel (the number of channels is listed on the top of each box) feature map. The spatial dimensions are maintained at 128 × 80 through the entire network

Our scatter estimation CNN (Fig. 2), similar to our previous network [16], took both the projected attenuation map (3D attenuation map projected to SPECT projection space via line integrals) and the scaled SPECT projection measurements individually as inputs and passed them to separate branches. Each of the input branches has three convolutional layers followed by a ReLU activation layer. The outputs of these two branches were then concatenated along the channel dimension and fed through three convolutional layers with ReLU activations. To generate the non-negative estimated scatter projection, a point-wise convolutional layer and an additional ReLU layer were applied at the end. To preserve the spatial dimensions, all convolutions, except for the final one, used a 3 × 3 kernel with zero padding of size 1 for each dimension. We trained the scatter estimation network by minimizing the pixel-wise mean square error (MSE) between the estimated and GT scatter projections from SIMIND, using the Adam optimizer [22] with 800 epochs and initial learning rate of 0.0001. We implemented the network in PyTorch and trained on NVIDIA V100 GPU.

### Stage II: OS-EM for SPECT reconstruction

OS-EM [23] is a widely used algorithm for performing 3D SPECT reconstruction that aims to estimate the image (emission distribution) ${\varvec{x}}$ from the noisy measurements (recordings of emitted particles) ${\varvec{y}}$, which are often statistically modeled as:

$$\user2{y }\sim {\text{Poisson(}}{\varvec{Ax}} + \overline{\user2{r}}{)}$$

where ${\varvec{A}}$ represents the system model, including ray-dependent factors such as attenuation and detector efficiency, and ${\varvec{r}}$ denotes the mean of background events such as scatter events. In this stage, we performed OS-EM reconstruction (number of subsets: 4, number of iterations: 16, matrix size: 128 × 128 × 80, voxel size in mm: 4.8 × 4.8 × 4.8) using our in-house MIRT program [24], with CT-based attenuation correction, CNN estimated scatter correction, as well as collimator-detector response compensation with no post-filtering. All reconstructed SPECT images were linearly interpolated and registered to CT image space afterward.

### Stage III: generating the absorbed dose-rate map with deep residual learning network

#### Datasets

The 12 virtual patient phantoms that had gone through the test phase of the scatter estimation CNN were used for training/testing the dosimetry network (6 for training and 6 for testing). To generate the GT dose-rate maps to use as the training labels, we first ran the in-house Monte Carlo dosimetry code (dose planning method, DPM [25]) with the true activity and density maps corresponding to the phantom as the input (Phantom + MC Dose). For DPM, we simulated approximately one billion histories after considering statistical uncertainty. With this number of histories, the relative uncertainty in absorbed dose-rate was < 0.1% at organ and lesion level and < 1% at the voxel level for voxels within these structures. For use in our residual learning network described below, we also generated dose-rate maps by DVK convolution, where the 90Y kernels in water (1.0 g/cm^3^) were generated by DPM MC. To include all possible events, the beta particle kernel size was designed to be 23 × 23 × 13 (with voxel size 0.98 × 0.98 × 2 mm^3^) given the fact that the maximum range of 90Y beta particles in tissue is about 11 mm. SPECT reconstructions obtained from stage II were convolved with DVKs with fast Fourier transform (FFT). Density scaling was performed by dividing each voxel by the corresponding density value (g/cm^3^). To avoid the unreasonably high dose-rate estimation in extreme low-density regions, the DVK dose-rate with corresponding voxel density lower than 1.0 g/cm^3^ was set to 0. Such a cutoff was also used within DPM.

#### Network

**Fig. 3:**
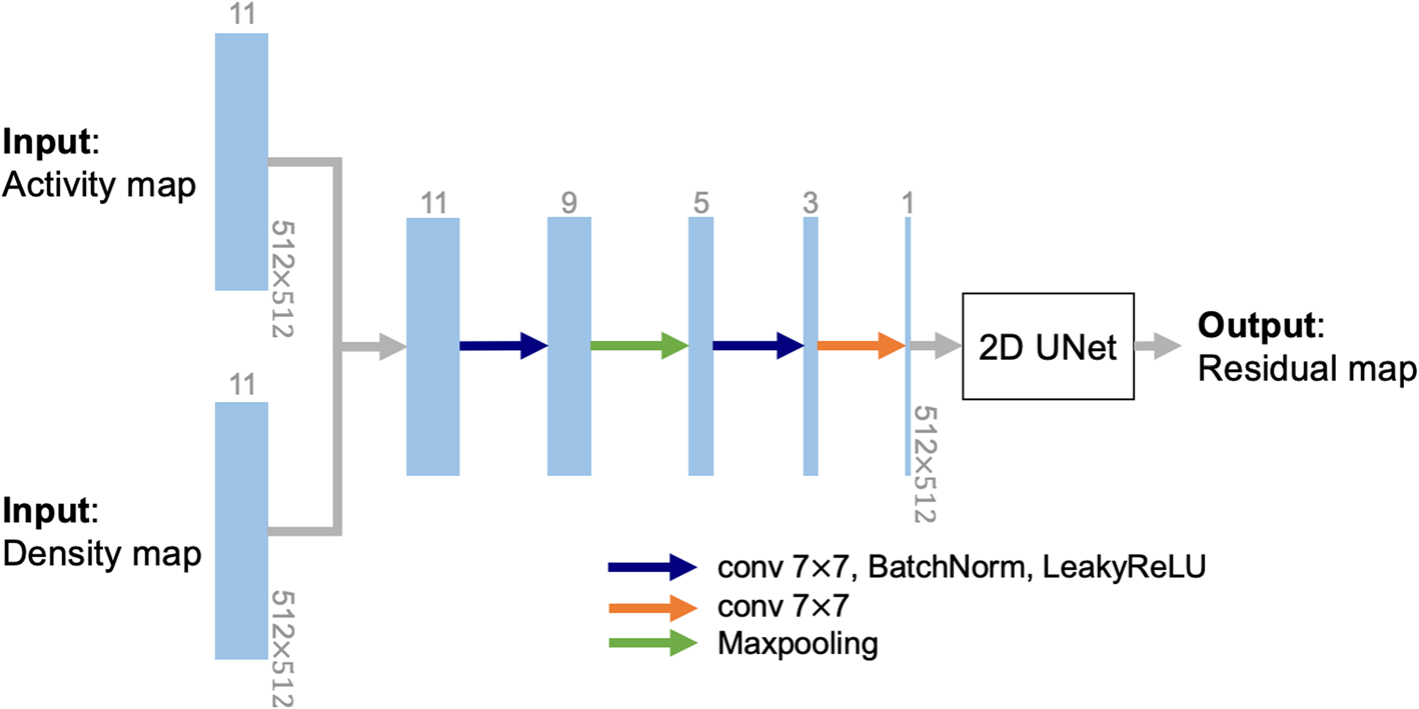
Architecture of our residual learning network (DblurDoseNet) for SPECT-based absorbed dose-rate map generation. Each blue box corresponds to a multi-depth (the depth is listed on top of each box) feature map. The depths of kernels in the three 3D-convolution operations are 5, 5, 3, respectively. The input size was 512 × 512 × 11 with 11 adjacent slices to capture the dose contributions from 90Y beta particles. The spatial dimensions are maintained at 512 × 512 in all layers

The network takes the reconstructed SPECT (from Stage II) and CT images as inputs to generate the dose-rate map with high resolution (deblurred). The network implemented in this stage (Fig. 3) for the proposed 90Y dose-rate estimation was inspired by our previous implementation (DblurDoseNet) for 177Lu [20], and we further extended and fine-tuned the hyperparameters. Here, we use the physics-based DVK convolution approach to obtain a fast initial estimate of the dose-rate map. Our network was designed to have a residual structure that learned only the subtle differences between the GT dose-rate maps from MC and DVK dose-rate maps. The inputs to our residual learning network consist of packs of scaled activity maps (SPECT images reconstructed with CNN scatter correction, scaled so that all voxels sum up to a normalizing constant for faster and more stable convergence during training) and density maps. The input size was 512 × 512 × 11 (with voxel size 0.98 × 0.98 × 2 mm^3^) with 11 slices packed to capture all possible dose contributions from 90Y beta particles. The input packs were concatenated along the channel dimension and passed to a 3D convolutional layer-based (kernels with spatial size of 7 and depth of 5, 3, 3, respectively) feature extractor and 2D feature maps corresponding to the middle slice of the inputs were generated. The 2D feature maps were then fed to a 2D U-Net. The corresponding DVK dose-rate maps (provided to the stage III as an initial estimation of dose-rate maps for residual learning) were added to the outputs of the 2D U-Net to generate the outputs of the stage III, as well as the outputs of the entire framework: estimated dose-rate maps (density correction and inversely scaling was applied at the last layer). The dose-rate unit in the network output is nGy/MBq-sec, aligning with the unit of the training labels (GT dose-rate maps) up to a scaling factor. The network was trained to minimize the pixel-wise MSE between the GT dose-rate maps (Phantom + MC Dose) and the estimated dose-rate maps (with residual learning) on a single NVIDIA V100 GPU (batch size: 32, optimizer: Adam with initial learning rate of 0.001, epochs: 300). The training/validation curves converged visually after ~ 10 h of training, as illustrated in Additional file 1: Fig. S4. Note that we trained two networks in stage I and stage III separately and sequentially, without backpropagating through the OS-EM.

### Ablation study

As an ablation of our framework, instead of two networks for scatter and dosimetry separately we also investigated training and fine-tuning only the stage III model (DblurDoseNet) to perform both tasks. The same 6 training cases used previously to train the DblurDoseNet were used here. In this case, the single-stage model took SPECT reconstruction with no SC and density maps as inputs and used the DVK dose-rate map (FFT convolved DVK with SPECT w/no SC) for residual learning.

### Metrics used for evaluation

Performance was evaluated for the total image and both the lesion and the healthy liver (treated liver minus the lesions) volumes of interest (VOIs) in the phantoms. Qualitatively, results were compared by visual assessment of images and line profiles. Quantitatively, the following metrics were used. Let $n_{p}$ denotes the total number of voxels in the VOI. The GT and estimated images are denoted by ${\varvec{x}}$ and $\hat{\user2{x}}$, respectively.

*Normalized mean absolute error (NMAE)* is defined as:

$${\text{NMAE}} \triangleq \frac{{\left| {\frac{1}{{n_{p} }}\left( {\mathop \sum \nolimits_{i = 1}^{{n_{p} }} {\varvec{x}}_{i} - \mathop \sum \nolimits_{i = 1}^{{n_{p} }} \widehat{{{\varvec{x}}_{{\varvec{i}}} }}} \right)} \right|}}{{\frac{1}{{n_{p} }}\mathop \sum \nolimits_{i = 1}^{{n_{p} }} {\varvec{x}}_{i} }} \times 100\%$$

*Normalized root mean squared error (NRMSE)* is defined as:

$${\text{NRMSE}} \triangleq \frac{{\sqrt {\frac{1}{{n_{p} }}\mathop \sum \nolimits_{i = 1}^{{n_{p} }} \left( {{\varvec{x}}_{i} - \widehat{{{\varvec{x}}_{i} }}} \right)^{2} } }}{{\sqrt {\frac{1}{{n_{p} }}\mathop \sum \nolimits_{i = 1}^{{n_{p} }} {\varvec{x}}_{i}^{2} } }} \times 100\%$$

The subscription indicates the *i*th voxel of the object.

We evaluated the CNN scatter projections using NRMSE relative to the GT scatter projections from SIMIND. The SPECT reconstructions with no SC and the CNN estimated SC were evaluated using NRMSE relative to the reconstructions with the true scatter estimates from SIMIND as the GT. The dose-rate maps were evaluated using both NMAE and NRMSE relative to the GT dose rate maps directly from the phantom (Phantom + MC Dose). We compared performance of dose-rate maps corresponding to (1) SPECT reconstruction with CNN scatter correction and DVK convolution (SPECT w/CNN SC + DVK) (2) SPECT reconstruction with CNN scatter correction and DPM MC dosimetry (SPECT w/CNN SC + MC Dose) and (3) SPECT reconstruction with CNN scatter correction and deep learning dosimetry (SPECT w/CNN + DblurDoseNet). All dosimetry evaluations were performed on dose-rate maps (output of the framework) instead of dose maps. For 90Y-RE, where there is no re-distribution of activity, conversion of dose-rate maps to dose-maps simply requires scaling by a constant factor, which is the time integral of the mono-exponential decay curve with a half-life equal to physical decay.

### Torso phantom measurement

The torso phantom comprised a liver section filled with water, lung compartments filled with Styrofoam beads and water (simulating lung tissue density), and a spine insert for bone density. The liver had a volume of 1200 mL and incorporated three lesion inserts: a 29 mL ovoid (insert 1), a 16 mL sphere (insert 2), and an 8 mL sphere (insert 3). The phantom's total Y90 activity during imaging was 2.0 GBq. Activity concentrations were 6.4–7.8 MBq/mL for the liver inserts and 1.3 MBq/mL for the liver excluding inserts, aligning with clinical scenarios for Y90 RE. SPECT/CT acquisition lasted 30 min, ensuring a count level (9 million counts) consistent with standard patient studies. The ground truth activity map was generated by masking the CT image and assigning the true (uniform) activity concentration to each compartment.

### Patient studies

90Y SPECT/CT images from 4 clinical patients (distinct from the patients used to generate virtual patient phantoms) were selected to represent clinical application. Patients covered diverse scatter conditions and dose-rate levels with injected activities ranging from 1.3 to 4.0 GBq. 90Y SPECT/CT scans were acquired with a duration of approximately 30 min within a few hours following the RE procedure on the same SPECT/CT system described previously. A total of 6 lesions (volume ranging from 4 to 59 mL) were segmented by the radiologist in these 4 patients. As in the phantom testing, patient measured SPECT projections and CT-based projected attenuation map were used to estimate scatter projections (Stage I) that were needed for OS-EM SPECT reconstruction (Stage II). DVK dose-rate maps (based on SPECT w/CNN SC) were produced, and packs of upstream SPECT images with CNN SC and density maps were subsequently input to the residual learning network to generate dose-rate maps (Stage III). In the absence of GT, to evaluate the dose-rate map of clinical patients, we compute line profiles and the lesion-to-background ratios. Here, the background VOIs were defined nearby in a uniform region of the liver with equivalent sizes and shapes to those of the target lesions.

## Results

### Testing in virtual patient phantoms

#### Evaluation on scatter projections and reconstructed SPECT

**Fig. 4:**
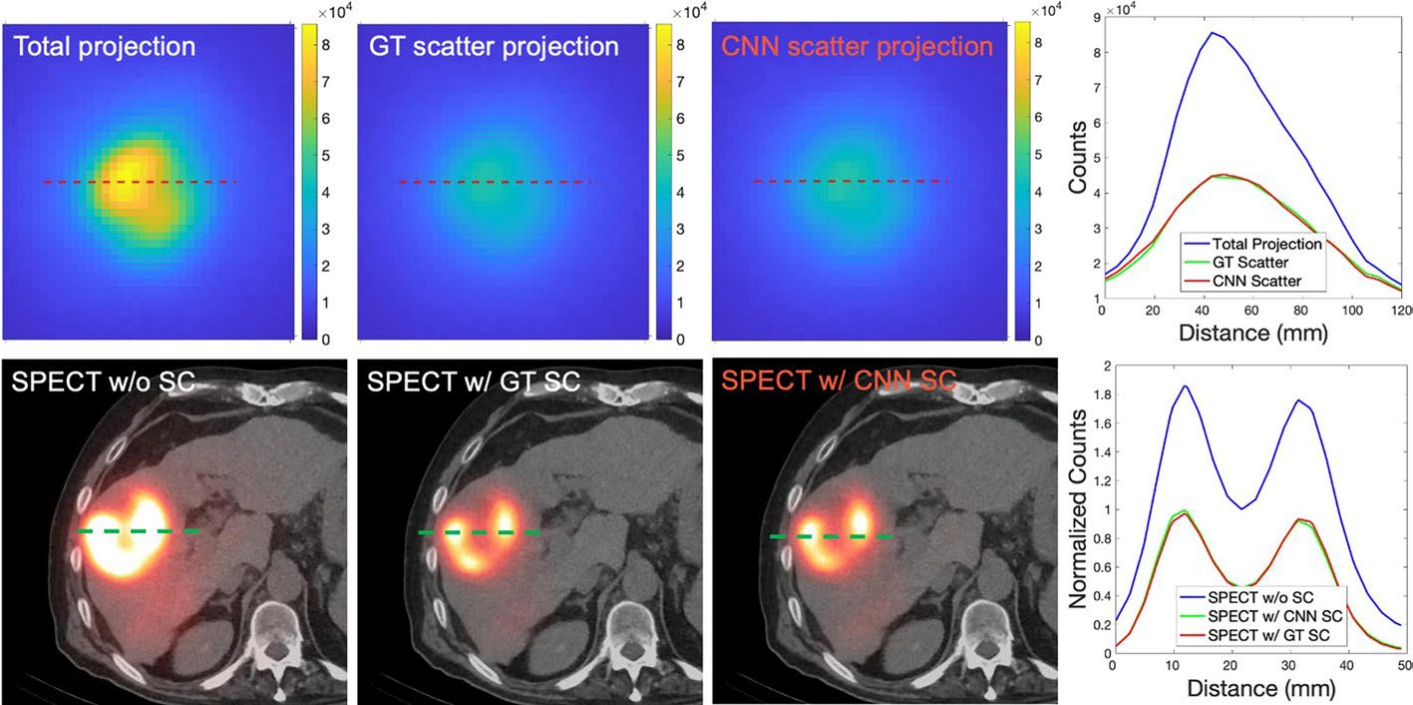
Comparison of SPECT scatter projections and reconstructed images for an example virtual patient phantom. The first row displays a projection view and line profiles of the total projection, the true scatter projection, and the CNN estimated scatter projection. The second row illustrates the SPECT reconstruction without scatter correction, SPECT reconstructions with GT and CNN estimated scatter correction, and their respective line profiles

Visually, the CNN estimated scatter projections were close to GT scatter projections from SIMIND. SPECT w/CNN SC also shows close agreement to SPECT w/GT SC, while SPECT w/o SC shows poor contrast and overestimation of counts. Figure 4 provides example images and line profiles. Table 1 reports the NRMSE of the estimated scatter projections (average 4.0% ± 0.7%), as well as the NRMSE of SPECT w/CNN SC (average 9.2% ± 1.7%) and SPECT w/o SC (average 149.5% ± 31.2%) relative to SPECT w/GT SC. The overall improvement was 94% across all tested virtual patients in terms of NRMSE of SPECT reconstruction.

**Table 1:** NRMSE of estimated scatter projections relative to the GT scatter projections from SIMIND MC as well as NRMSE of SPECT w/CNN SC and SPECT w/o SC, relative to SPECT w/GT SC

Virtual patient	#1	#2	#3	#4	#5	#6	#7	#8	#9	#10	#II	#12
*NRMSE (%)*												
Scatter projections	3.0	4.3	4.0	4.9	3.4	3.7	5.0	3.6	4.6	4.3	4.4	2.8
SPECT recon w/CNN SC	**11.1**	**9.5**	**11.5**	**8.9**	**7.5**	**9.6**	**7.4**	**9.0**	**12.6**	**8.1**	**7.0**	**8.6**
SPECT recon w/o SC	171.9	127.1	177.5	132.2	106.2	166.0	145.3	130.6	229.0	148.7	128.8	131.1

Mumbers in bold represent the best outcomes among the comparisons made within each table

Values presented are over the total image of the 12 virtual patients used for testing the scatter correction

#### Evaluation on dose-rate maps

**Fig. 5:**
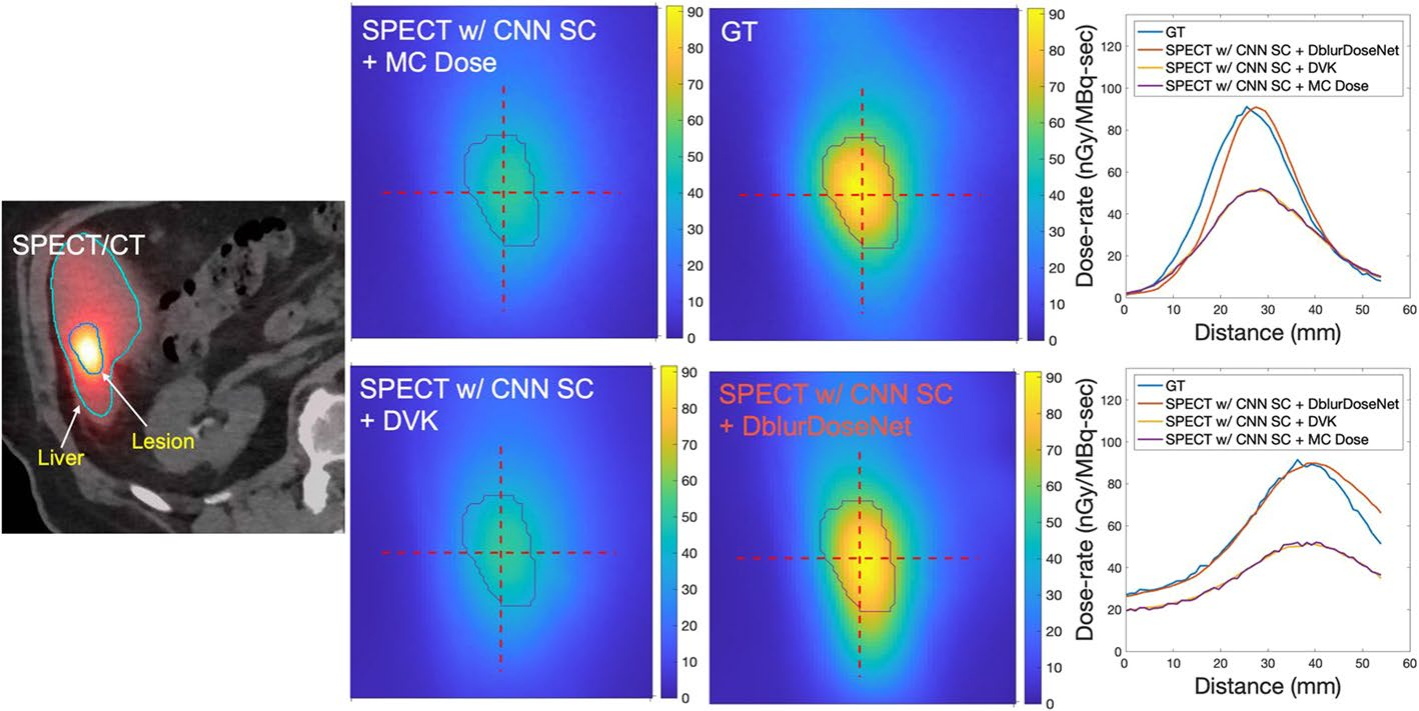
An example slice of SPECT/CT, along with dose-rate maps of a virtual patient with our proposed framework and MC and DVK dosimetry methods starting with the SPECT reconstruction with CNN SC. The upper right figure displays horizontal profiles, while the bottom right one illustrates vertical profiles

Visually, the dose-rate maps generated from the proposed framework were sharper and closer to the GT dose-rate maps, compared to DVK dose-rate maps and MC dose-rate maps. See Fig. 5, for example, images and line profiles. Table 2 reports the NMAE and NRMSE for mean dose-rate in lesions and healthy livers across all 6 test phantoms. Our framework, SPECT w/CNN SC + DblurDoseNet achieved a NMAE of 8.6% ± 5.7% and 5.4% ± 4.8% for dose-rate in lesions and healthy livers, respectively, while the corresponding NMAE values for SPECT w/CNN SC + MC Dose were 24.0% ± 6.1% and 17.7% ± 2.1%. For NRMSE of dose-rate in lesions and healthy livers, respectively, SPECT w/CNN SC + DblurDoseNet achieved 20.1% ± 5.0% and 26.3% ± 4.1%; whereas SPECT w/CNN SC + MC Dose achieved 27.6% ± 5.7% and 28.1% ± 5.0%. Over all phantoms, the improvement in results for our framework over MC was 66% for NMAE and 20% for NRMSE. Figure 7 provides cumulative dose-rate volume histograms corresponding to DVK, MC Dose, CNN and the GT dose-rate maps of virtual patients demonstrating more accurate voxel-level dose metrics, such as minimum dose rate to 10% (D10) and 90% of volume (D90), with our method compared with MC and DVK dosimetry.

**Table 2:** NMAE and NRMSE of dose-rate in lesions and healthy livers across 6 test virtual patients, using our framework, and MC and DVK voxel-dosimetry methods

	NMAE (%)			NRMSE (%)		
	SPECT w/CNN SC + DblurDoseNet	SPECT w/CNN SC + DVK	SPECT w/CNN SC + MC Dose	SPECT w/CNN SC + DblurDoseNet	SPECT w/CNN SC + DVK	SPECT w/CNN SC + MC Dose
*Virtual patient #I*						
Lesion I (932.1 mL)	**6.2**	18.3	18.3	27.5	26.3	**26.3**
Lesion 2 (41.8 mL)	**3.0**	24.1	24.0	**22.3**	28.7	28.6
Healthy liver	**4.1**	18.5	18.4	**33.6**	36.7	36.7
*Virtual patient #2*						
Lesion I (183.8 mL)	**12.2**	24.9	24.8	**21.4**	28.6	28.5
Healthy liver	**0.7**	17.0	17.0	**28.5**	30.8	30.8
*Virtual patient #3*						
Lesion I (56.9 mL)	**6.5**	21.0	21.0	**12.7**	22.4	22.3
Healthy liver	**5.1**	17.5	17.5	**23.5**	25.8	25.8
*Virtual patient #4*						
Lesion I (29.6 mL)	**12.4**	17.6	17.5	25.3	23.1	**23.1**
Lesion 2 (27.4 mL)	**7.3**	26.2	26.2	**18.1**	28.2	28.2
Lesion 3 (21.0 mL)	**10.5**	31.6	31.6	**17.6**	34.4	34.4
Lesion 4 (5.7 mL)	**21.7**	38.7	38.7	**23.7**	40.4	40.3
Healthy liver	**1.7**	16.6	16.6	**23.6**	24.6	24.6
*Virtual patient #5*						
Lesion I (26.4 mL)	**8.8**	25.7	25.7	**18.0**	31.4	31.3
Healthy liver	**14.2**	21.4	21.6	**24.0**	27.7	27.7
*Virtual patient #6*						
Lesion I (21.2 mL)	**2.8**	20.3	20.2	**15.4**	21.2	21.1
Lesion 2 (22.7 mL)	**0.6**	19.2	19.2	26.0	22.9	**22.9**
Lesion 3 (21.7 mL)	**11.6**	20.8	20.7	**13.5**	23.8	23.7
Healthy liver	**6.5**	16.3	16.4	25.0	24.2	**24.1**

Mumbers in bold represent the best outcomes among the comparisons made within each table

### Testing in torso phantom measurement

**Fig. 6:**
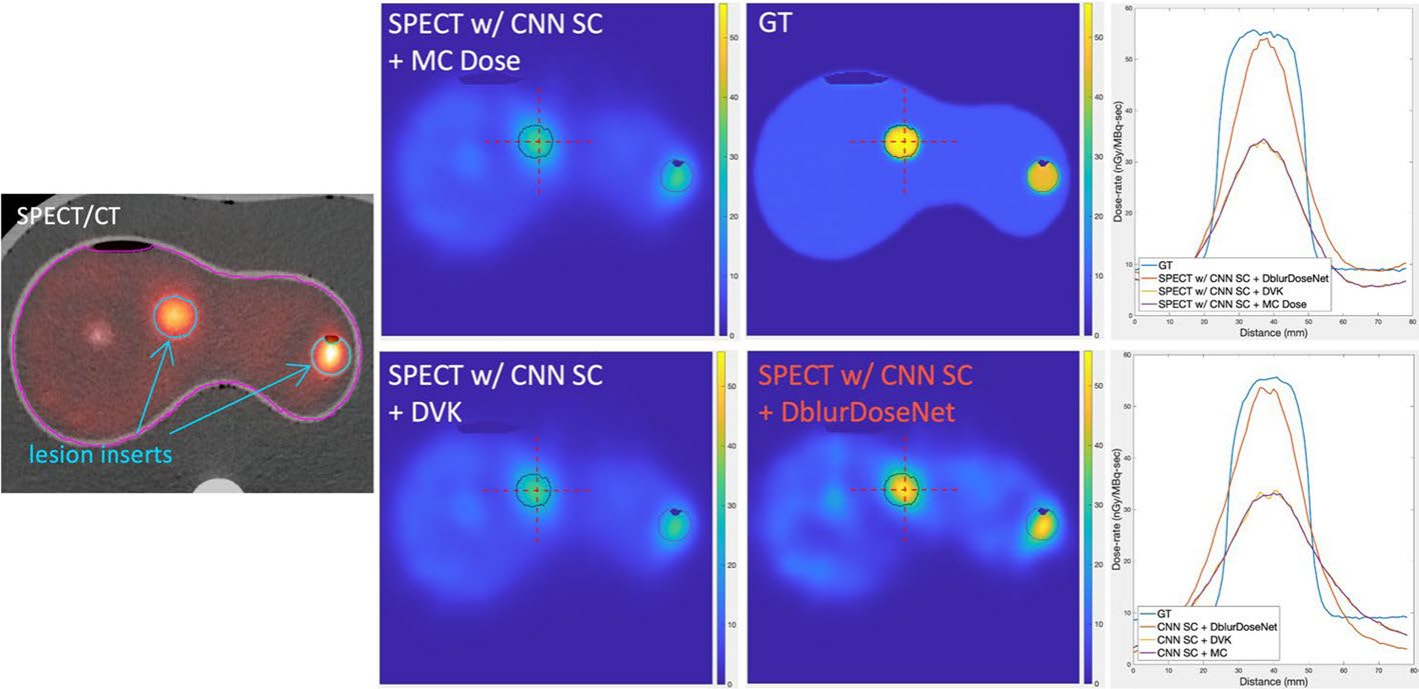
An example slice of SPECT/CT, as well as dose-rate map of the torso phantom with our proposed framework and MC and DVK dosimetry methods starting with the SPECT reconstruction with CNN SC, and line profiles across the center of the tumor insert (16 mL)

Figure 6 compares dose-rate maps of the torso phantom corresponding to our framework and with MC/DVK dosimetry methods. Visually, our framework, SPECT w/CNN SC + DblurDoseNet generated the dose-rate map with sharper lesion boundaries and closer to the GT. The proposed method achieved a significant lower NMAE for dose-rate in all lesion inserts, as shown in Table 3.

**Table 3:** NMAE and NRMSE of dose-rate in all lesion inserts of the torso phantom, using our framework, and MC and DVK voxel-dosimetry methods

	NRMSE (%)			NMAE (%)		
	29 mL ovoid	16 mL sphere	8 mL sphere	29 mL ovoid	16 mL sphere	8 mL sphere
SPECT w/CNN SC + DblurDoseNet	36.4	**29.9**	**19.3**	**9.2**	**18.4**	**10.4**
SPECT w/CNN SC + DVK	**29.2**	41.5	41.0	22.0	40.4	40.9
SPECT w/CNN SC + MC Dose	29.3	41.6	41.4	21.8	40.2	40.2

Mumbers in bold represent the best outcomes among the comparisons made within each table

### Application in patients

Figure 8 compares dose-rate maps of a representative clinical patient generated with our framework and with MC dosimetry. Compared with MC, the dose-rate map from our framework demonstrates sharper lesion boundaries. Table 4 provides lesion-to-background ratios for the mean dose-rate for the two methods. The proposed method achieves higher ratios (16.5% ± 6.7%) compared to MC dosimetry (12.4% ± 5.5%).

**Table 4:** Lesion-to-background ratio in mean dose-rate across all 4 test clinical patients, using our framework and MC dosimetry based on the CNN scatter-corrected SPECT reconstruction

	SPECT w/CNN SC + DblurDoseNet	SPECT w/CNN SC + MC dose
*Clinical patient #1*		
Lesion 1 (15.7 mL)	**24.1**	22.1
Lesion 2 (4.2 mL)	**23.0**	12.3
*Clinical patient #2*		
Lesion 1 (59.1 mL)	**13.2**	12.2
*Clinical patient #3*		
Lesion 1 (22.9 mL)	**11.8**	7.56
*Clinical patient #4*		
Lesion 1 (11.0 mL)	**19.5**	13.6
Lesion 2 (4.6 mL)	**7.6**	6.8

Mumbers in bold represent the best outcomes among the comparisons made within each table

### Results for ablation study

We compared the performance of this single-stage model versus the proposed framework with the same 6 test cases used previously. The single network approach showed comparable dosimetry performance to our proposed framework, with NMAE: 8.1% versus 8.6% and NRMSE: 17.4% versus 22.3%, respectively, on average across all test cases.

### Time cost

The data generation and training processes for our framework can be time-consuming and computationally expensive; however, these steps are only required to be performed once. The times listed here correspond to the Intel Core i9 @ 2.3 GHz CPU and NVIDIA V100 GPU. With regard to generation of datasets, it took ~ 16 h to generate all 128 MC-simulated SPECT projections (matrix size 128 × 128) by SIMIND for one digital phantom on the CPU with 16 processors. Generating the GT dose-rate map from the true activity/density map of a virtual patient (matrix size: 512 × 512 × 194) with DPM MC took ~ 1 h on the CPU using 1 processor. The scatter estimation network took ~ 12 min to train and can generate all (128) scatter projections for a test case within 1 s on a single GPU. In the final stage, it took ~ 10 s to generate the DVK dose-rate map on CPU, and the residual learning network required ~ 10 h to train and could estimate the dose-rate map in ~ 20 s on a single GPU.

## Discussion

To our knowledge, this is the first study reporting 90Y SPECT-based dosimetry using deep learning methods for both scatter estimation and voxel dosimetry. The proposed method offers the advantage that it generates dose-rate maps using a framework that was trained by the true activity map devoid of scatter and resolution effects/artifacts. This is distinct from previous reports on dosimetry with deep learning for diagnostic and therapy applications where the GT images for training were generated from SPECT or PET [17–19]. Our approach mitigates the impact of poor SPECT image quality on dosimetry and the accuracy–efficiency trade-off of MC-based scatter estimation and voxel dosimetry methods. Our framework is especially well-suited for 90Y SPECT-based dosimetry, which is impacted by the complexities of bremsstrahlung imaging.

**Fig. 7:**
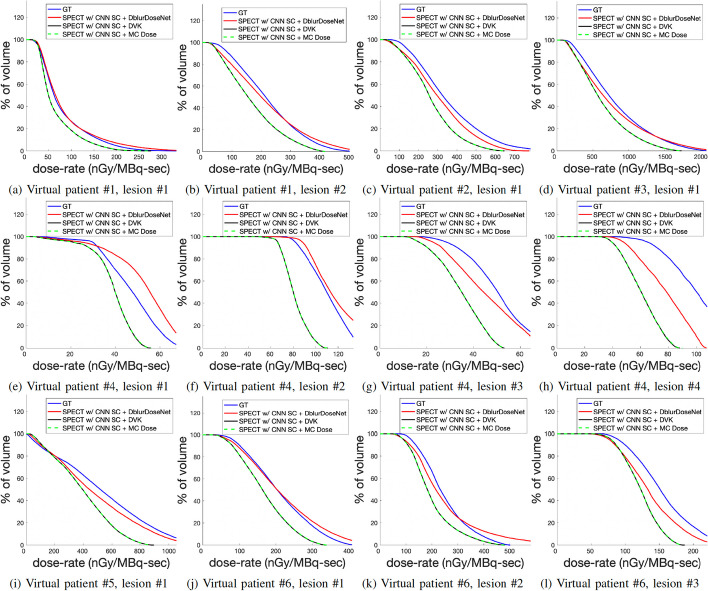
Lesion-specific cumulative dose-rate volume histograms (DRVH) corresponding to the proposed framework and MC/DVK dosimetry methods starting with the SPECT reconstruction with CNN SC

**Fig. 8:**
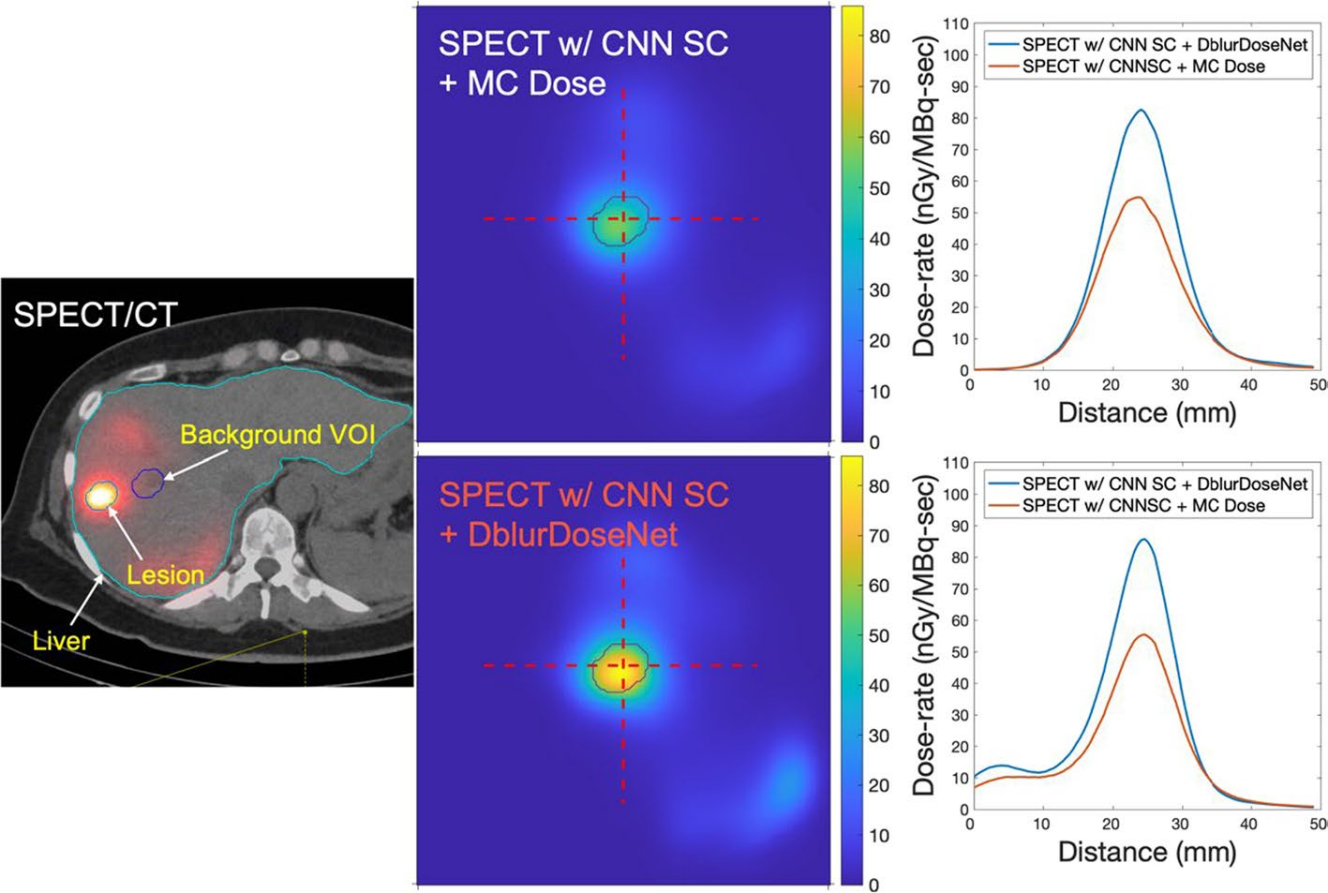
An example slice of SPECT/CT, along with dose-rate maps of a representative clinical patient using the proposed framework and MC dosimetry, are presented. Background VOI is delineated to compute the lesion-to-background ratio (as shown in Table 4). The upper right figure displays horizontal profiles, while the bottom right one illustrates vertical profiles

The improved dosimetry with our network is evident in Figs. 5, 6, 7 and 8 where non-learning voxel dosimetry methods fail to generate accurate dose-rate maps. We demonstrated mean dose-rate errors of only 8.6% on average for lesions and 5.4% on average for healthy liver with a maximum of 21.7% across a range of virtual patients (Table 2). The dose-rate error primarily stems from the DVK dose-rate maps’ quality that is contingent on the SPECT images that have limited resolution. Partial Volume Effects associated with spatial resolution impact SPECT quantification and can depend on various factors, such as object size, shape, and activity distributions [26, 27]. In addition to SPECT resolution effects, the test data might encompass unique features absent from the training set, making it challenging to always achieve optimal recovery results. Overall, the improvement with our framework over MC dosimetry was 66% in terms of NMAE and 20% in terms of NRMSE. Due to the lack of ground truth dose-rate maps in the case of clinical patients, our quantitative evaluations had to be limited to virtual patient phantoms. However, we conjecture that the sharper lesion boundaries (Fig. 8) and enhanced lesion-to-background ratios (Table 4) in dose-rate maps of clinical test cases are indication of increased dosimetry accuracy with the proposed framework.

To avoid overfitting, we used independent data for training/validating/testing the two networks and for testing the overall framework. Both the neural networks in stage I and stage III were designed to follow commonly used architectures in medical imaging. As a result, these two networks both used only modest amounts of training data, but the framework can still provide promising results as determined by metrics applied to each stage. Due to the presence of the OS-EM reconstruction algorithm in stage II, the entire framework was not trained as a single unit using backpropagation algorithms end-to-end. Therefore, we trained the two neural networks in the framework separately using backpropagation, since it would be too computationally expensive to backpropagate through all the OS-EM iterations to jointly optimize all components of the framework. Future works include comparing with a fully end-to-end training method that backpropagates through the OS-EM, where the scatter correction network and the dosimetry network are jointly optimized [28].

In the third stage of our proposed framework, the DblurDoseNet was designed to learn the residual between the GT and the DVK dose-rate map. Specifically, since the DVK dose-rate map is computed from SPECT reconstructed images, this stage effectively learns to counteract the imperfections inherent in the reconstruction process, primarily focusing on deblurring (hence the name DblurDoseNet). This suggests its potential to revert the blurred dose-rate map back to its original one (corresponding to the activity map devoid of scatter and blurring), whether from a clinical patient scan, a virtual patient phantom, or a high-resolution digital phantom such as XCAT [29]. The reason we trained the framework with SPECT-derived virtual patient phantoms is because our evaluations revealed that when the framework were trained using piece-wise linear activity maps such as in XCAT phantoms, it resulted in unnaturally uniform dose-rate maps when testing on patient data. This discrepancy is attributed to the divergent data distributions between training and testing phases. For instance, virtual patient phantoms display non-uniform (and more clinically representative) activity distributions, whereas XCAT phantoms have a uniform (and less representative distribution). It is generally assumed and preferred that the training and testing data exhibit similar distributions. One direction for future research could be to develop new network architectures that can adeptly learn from both types of distributions. A limitation of this study is the training on a relatively small dataset, potentially affecting the framework’s robustness on novel test data. However, given the promising results achieved with our current set of virtual patient phantoms, we predict that the framework would perform consistently and generalize well if it is exposed to a broader range of Y90 SPECT data features.

The single-stage model provides a slightly faster workflow (saving about 1 s of computation time that is needed for separately estimating scatter in our framework) than the proposed method. Furthermore, separately training two networks may require more training data than training a single network, which can be considered as another limitation of our approach. For example, our proposed framework used 6 training cases for scatter network and 6 for the dosimetry network. In contrast, one could use all 12 training cases simultaneously for training and validating a single-stage model. An advantage of the proposed framework is that it generates both an accurate SPECT reconstruction, and a dose-rate map unlike the single-stage network, which generates only the dose-rate map. Having access to the higher quality SPECT image with scatter correction for diagnostic purposes in addition to the dose-rate map enhances clinical relevance.

## Conclusion

In this work, we proposed a unified deep learning framework for 90Y bremsstrahlung SPECT/CT scatter estimation and voxel-level dosimetry, starting from the SPECT projections and CT image. The framework consists of three stages: CNN-based scatter estimation, SPECT reconstruction with SC, and absorbed dose-rate map generation with our residual learning network trained using true activity maps devoid of scatter and resolution effects. Across testing and evaluating on multiple VOIs of a series of clinically relevant virtual patient phantoms, our proposed method demonstrated higher estimation accuracy than the current “gold-standard” MC voxel dosimetry method and enjoys substantially faster computing speed. Furthermore, in clinical studies, we observed sharper dose-rate maps generated by our framework, which we conjecture corresponds to higher accuracy. Our framework's ability to enhance both computing speed and accuracy is of much significance for clinical dosimetry following 90Y-RE.

### Supplementary Information

**Additional file 1. Supplementary Figures and Tables.** This file includes Supplemental Figures 1, 2, 3, and 4, and Supplemental Table 1. The Supplemental Figures 1, 2, 3 and Table 1 provide detailed visualizations of the virtual patient phantoms, and the Supplemental Figure 4 illustrates the convergence of training/validation curves.

## Data Availability

The datasets generated during and/or analyzed during the current study are available from the corresponding author on reasonable request. Code for reproducing the results will be available at https://github.com/umjiayx/spect0 after the paper is accepted.
