# Supplementary material for: 90Y SPECT scatter estimation and voxel dosimetry in radioembolization using a unified deep learning framework
Source: EJNMMI Phys. 2023 Dec 13;10:82. doi: 10.1186/s40658-023-00598-9 (PMC10719178; doi:10.1186/s40658-023-00598-9)
Supplement: Supplementary file 1 — Additional file 1. Supplementary Figures and Tables. This file includes Supplemental Figures 1, 2, 3, and 4, and Supplemental Table 1. The Supplemental Figures 1, 2, 3 and Table 1 provide detailed visualizations of the virtual patient phantoms, and the Supplemental Figure 4 illustrates the convergence of training/validation curves. [file 40658_2023_598_MOESM1_ESM.docx]

**Supplemental Table 1**: A demographic table of all patients included in the study, including the age, BMI, gender specifications.

|  | **Sex** | **Height (m)** | **Weight (kg)** | **Age (Y)** |
| --- | --- | --- | --- | --- |
| **Treatment 1** | M | 1.765 | 73.936 | 70 |
| **Treatment 2** | M | 1.778 | 120.203 | 76 |
| **Treatment 3** | M | 1.727 | 90.719 | 59 |
| **Treatment 4** | M | 1.702 | 87.544 | 53 |
| **Treatment 5** | M | 1.778 | 105.000 | 77 |
| **Treatment 6** | F | 1.676 | 59.875 | 68 |
| **Treatment 7** | F | 1.626 | 81.647 | 69 |
| **Treatment 8** | M | 1.778 | 79.379 | 62 |
| **Treatment 9** | M | 1.753 | 87.100 | 66 |
| **Treatment 10** | M | 1.753 | 87.100 | 66 |
| **Treatment 11** | M | 1.829 | 108.863 | 66 |
| **Treatment 12** | M | 1.778 | 95.254 | 62 |
| **Treatment 13** | M | 1.676 | 84.369 | 79 |
| **Treatment 14** | M | 1.726 | 92.126 | 66 |
| **Treatment 15** | M | 1.708 | 86.183 | 74 |
| **Treatment 16** | M | 1.778 | 105.000 | 77 |
| **Treatment 17** | M | 1.753 | 90.719 | 66 |
| **Treatment 18** | F | 1.636 | 81.647 | 65 |

**Supplemental Fig. 1**: Example slices for virtual patient phantoms (used for training stage I) to show the variability.


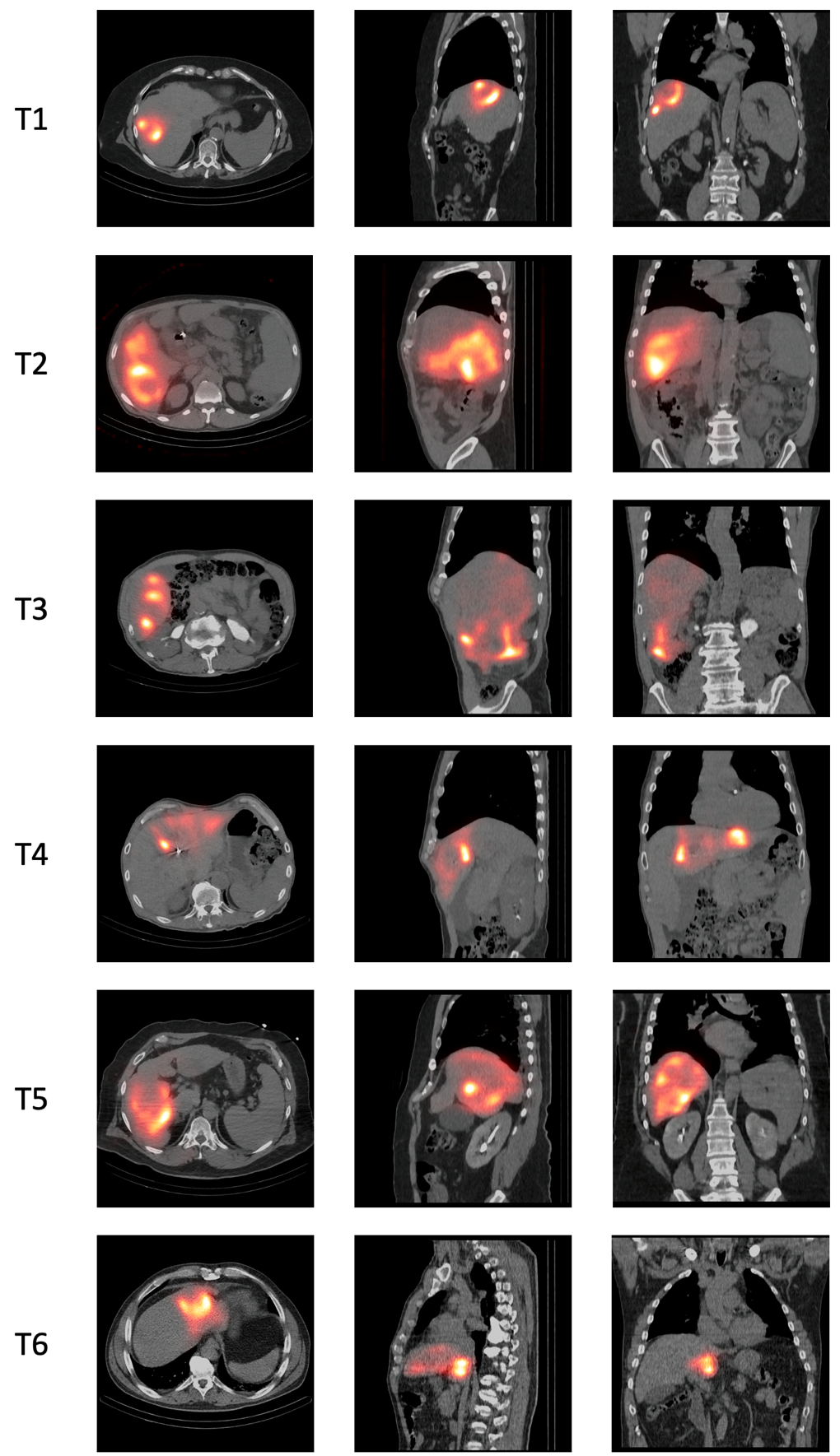


**Supplemental Fig. 2**: Example slices for virtual patient phantoms (used for testing stage I and training stage III) to show the variability.


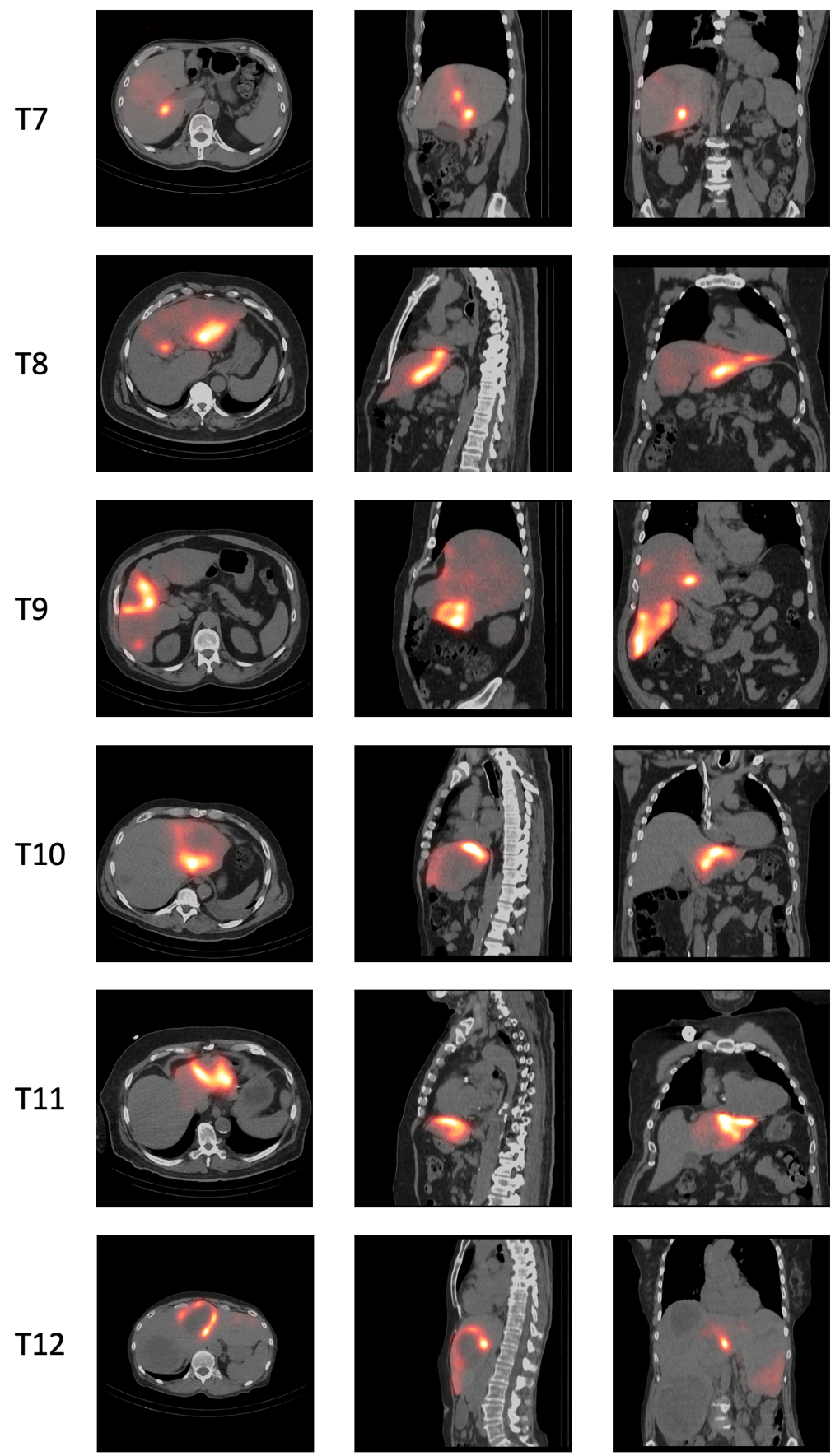


**Supplemental Fig. 3**: Example slices for virtual patient phantoms (used for testing stage III and the framework) to show the variability.


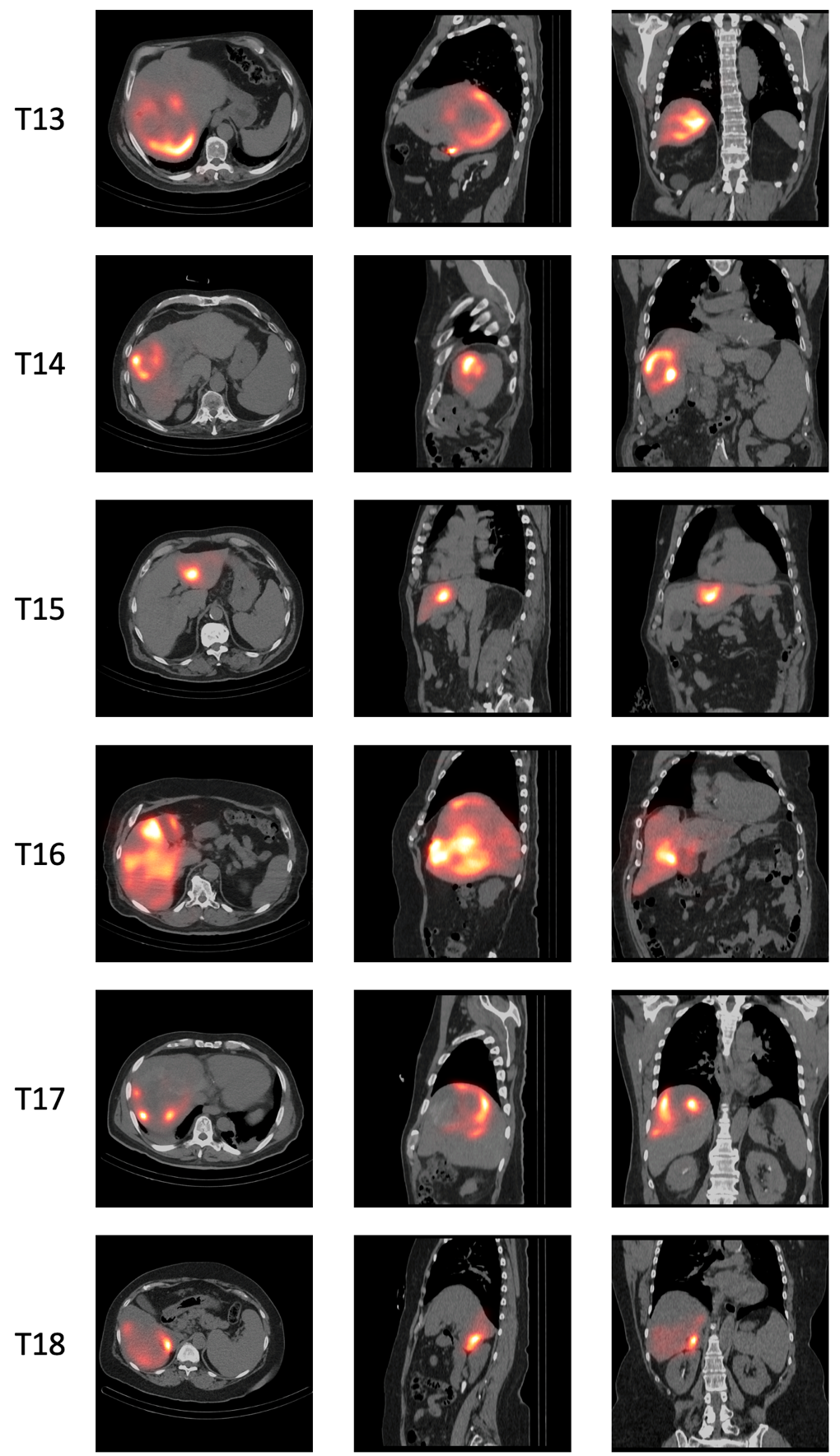


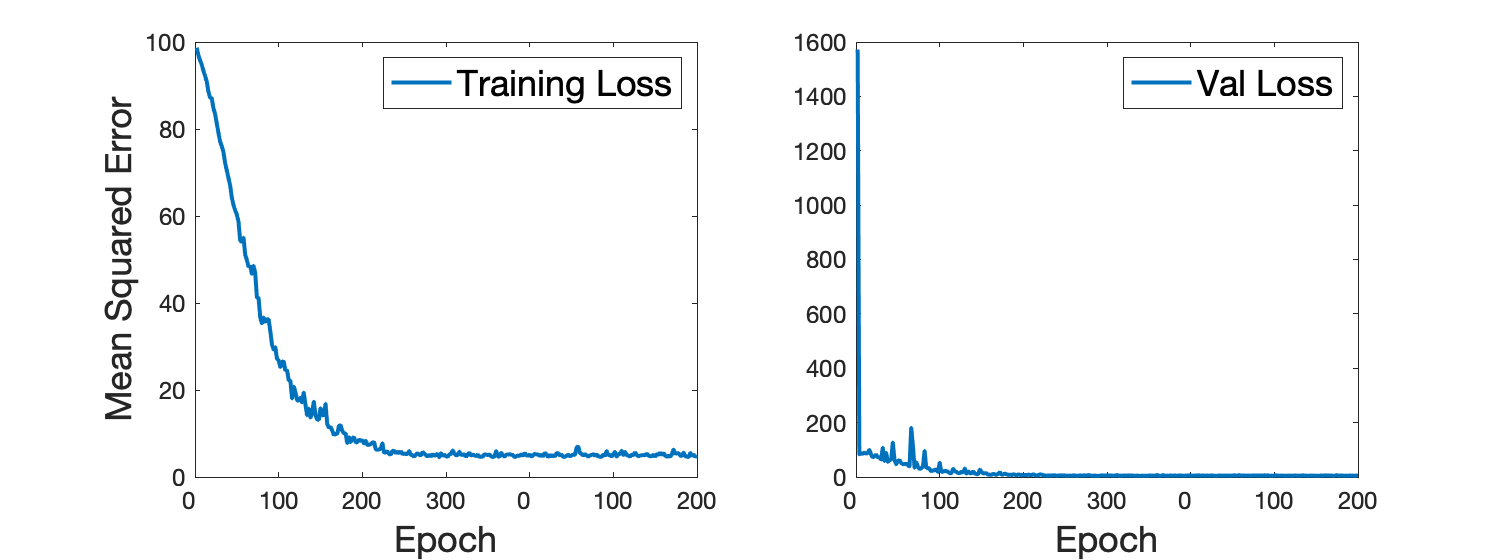


**Supplemental Fig. 4**: Learning curves (training loss and validation loss) converged visually when training the deep residual neural networks in stage III.
